# Importance of livestock diseases identified using participatory epidemiology in the highlands of Ethiopia

**DOI:** 10.1007/s11250-019-02187-4

**Published:** 2020-01-03

**Authors:** Solomon Gizaw, Hiwot Desta, Biruk Alemu, Azage Tegegne, Barbara Wieland

**Affiliations:** International Livestock Research Institute, PO Box 5689, Addis Ababa, Ethiopia

**Keywords:** Participatory epidemiology, Diseases, Livestock, Gender, Smallholders

## Abstract

Livestock are a major cornerstone for livelihoods of smallholders in the highlands of Ethiopia. However, infectious diseases are a key constraint in livestock production affecting productivity and food security. This study aimed to understand livestock producers’ perception on importance and epidemiology of livestock diseases. Participatory tools using focus group discussions were used to identify and rank livestock species, major livestock diseases, and their epidemiological patterns in smallholder systems. The study covered 17 districts in four regions, with two or three *kebeles* (smallest administrative unit) included in each district. To capture views of women and men, separate focus group discussions (FGD) were conducted. Data collected through proportional piling were used in generalized linear model analysis. Taking sheep as a reference, cattle were significantly the most preferred species with the odds of cattle scoring higher than sheep being 2.08 times (*P* = 0.000). Sheep and poultry were equally the second most important livestock, followed by goat and equine. There were no statistically significant differences between men and women FGD groups and between the four regions in their preference for livestock species. Twelve out of 28 identified livestock diseases were mentioned by at least 10% of FGDs in their list of five most important diseases. Blackleg, foot, and mouth disease, lumpy skin disease, anthrax, and bovine pasteurellosis were ranked as the top five diseases by both men and women focus groups. Reasons for high scores included suddenness of death, zoonosis risks, animal age groups and species affected, frequency of occurrence, rate of transmission in herds, curability, and inefficient vaccination. Seasonality and agro-ecology were also factors associated with disease occurrence. The study also documents that adult men and women are more involved in activities related to disease transmission compared with other family members. When asked about impacts of diseases, loss of livelihood, displacement, and infection with zoonoses were mentioned, with women considered equally affected as men. In conclusion, participatory tools allowed understanding disease priorities of and their perceived epidemiology in smallholder systems. The perceptions and priorities of men and women are very similar, and both need to be involved in designing health management interventions. Based on the findings of this study, the intervention strategies listed in the 2013 animal health strategy of Ethiopia (MoA and ILRI 2013) seem pertinent and need to be implemented to overcome the challenges of diseases.

## Introduction

Rural Ethiopia is home for 87% of the country’s 100 million people. The smallholder farmers in the highland mixed livestock-crop production system rely heavily on livestock for their livelihoods, livestock serving as source of cash income for incidental/daily expenses (poultry, egg and milk sale), big expenses (fattened cattle and sheep/goat), quality protein supplementing cereal-based diet, and power for crop farming. The country is endowed with diverse animal genetic resources and agroecology. However, the contribution of the livestock sector to household livelihoods and the national economy is well below potential (e.g., Aklilu et al. 2013). Livestock provides only 16% of the total GDP (equivalent to 30% of agricultural GDP) and generates 14% of the country’s foreign exchange earnings (CSA 2009).

Ethiopia is endemic to a number of livestock diseases, including OIE-listed diseases such as contagious bovine pleuropneumonia (CBPP), lumpy skin disease (LSD), foot and mouth disease (FMD), Newcastle disease (NCD), Peste des petits ruminants (PPR), sheep and goat pox (SGP), and African horse sickness (AHS) (NEPAD 2005). Besides affecting the production of livestock products, livestock diseases have other impacts in the highland mixed crop-livestock system, namely their impact on work performance of oxen which could lead to severe food insecurity and poverty. Mortality rates of up to 49% in oxen, 50% in local cows, 81% in crossbred cows, 52% in local calves/heifers, and 60% in crossbred calves/heifers have been reported in four regional regions in Ethiopia (Genbremedhin et al. 2017).

Livestock diseases reduce income and affect livelihoods of livestock keepers and jeopardize food security at local and national level. Despite the substantial export demand and the countries potential, the presence and prevalence of a number of trade-limiting transboundary livestock diseases has denied the country access to international market and makes it vulnerable to trade bans. A 2005 report (NEPAD 2005) estimated annual loss of about Birr 1.5–2.5 billion from the export market due to animal diseases. Diseases cause huge economic loss to producers and livestock exporters. A study of five cattle exporting enterprises with 4321 bulls kept for export at quarantines found infection rates of 12.9% for FMD and 8.0% for CBPP and estimated financial losses of about USD 241, 2341 due to the diseases between November 2013 and May 2014 (Birhanu 2014). Animal diseases also have an important impact on human health, with 60% of human infectious diseases being of animal origin. Ethiopia is endemic to a wide range of such zoonotic diseases (MoA and ILRI 2013).

The disease problem is rooted, among other causes, in limited understanding of the epidemiology of diseases in the various geographic regions, agroecological zones, and seasons. Lack of epidemiological information hampers development of disease control strategies. Epidemiological studies using conventional veterinary science methods have been limited due to the limitations in laboratory and logistic resources. A participatory approach, participatory epidemiology (PE), has been proven a suitable approach in such situations and has helped to improve understanding of diseases, and options for disease control through the systematic use of participatory methods (Catley et al. 2012). The approach is particularly valuable in the conditions of developing countries where smallholders’ production objectives and management practices vary and in areas where diverse agroecological and seasonal variations exist and enables to consider smallholders priorities and their indigenous knowledge in designing health interventions. The objective of this study was to understand livestock disease patterns in four highland regions of Ethiopia and the roles and indigenous knowledge of different household members including women and men household members using participatory epidemiology approach. The results of this study would be used to design effective disease control interventions across the different agro-ecologies and identify the roles of women and men farmers in implementing interventions.

## Materials and methods

### Sampling and data collection

The study was conducted in four regions Ethiopia, namely Amhara, Oromia, SNNPR (Southern Nations and Nationalities and Peoples region), and Tigray. In 17 districts, 37 *kebeles* (the smallest administrative unit) were selected; details on the agroecological characteristics are shown in Table 1. Two focus group discussions (FGD), each consisting of six to eight men and six to eight women members, were held in each *kebele*. However, men and women FGDs were not held in two and three of the *kebeles*, respectively, and two women FGDs were held in one of the *kebeles*, resulting in a total of 70 FGDs.

**Table 1:** Sampling structure for focus group discussions

Region	No. of districts	No. of *kebeles*	Agro-ecological zones	Altitude (m)	Rainfall (mm)	Temperature (°C)
Amhara	2	4	Moist highland	2636	1137	14.8
	2	4	Moist mid-highland	1912	1081	18.9
	2	5	Wet mid-highland	2023	1486	17.6
Oromia	1	3	Dry mid-highland	1761	837	19.5
	2	4	Moist highland	2583	1137	14.2
	2	4	Wet mid-highland	1892	1759	18.4
SNNPR	1	2	Moist highland	2588	1276	14
	1	3	Moist mid-highland	1948	1213	18
Tigray	1	2	Dry highland	2436	581	16.3
	3	6	Dry mid-highland	2036	612	18.6
Overall	17	37	–	–	–	–

Participatory tools were used to identify priority livestock species and diseases and their epidemiological patterns. Beans were used for proportional piling to elicit farmers’ priorities and perceptions. For this, participants were asked to allocate 100 beans according to preferences for alternative outcomes (livestock species and diseases). The FGDs were first asked to discuss and identify the five most important livestock species by allocating 100 beans to the five species. Then five most important livestock diseases were identified similarly and the local/traditional name of each disease or syndrome, and its clinical signs were described by the FGD groups, which were used by the local veterinarians who facilitated the FGDs to translate the diseases/disease syndromes into their equivalent scientific names. The seasonal distribution of each of the five diseases and the livestock age groups affected by the diseases were then discussed by dividing the scores for each disease into four seasons according to the importance of the disease in the four seasons. Effects of diseases on the household in general and household members individually and activities and household members involved in disease transmission were discussed and described, and contribution of each family member was scored out of 20 beans. Disease coping mechanisms were also discussed.

### Data analysis

A mixed-methods approach to data analysis was used. Qualitative information based on notes taken on the discussions during the FGDs was coded into categories, for example to define and group different diseases or reasons to prioritize diseases and livestock species. For quantitative information, descriptive statistics including proportions of FGD groups’ scorings for the different species and diseases were disaggregated by gender of the FGD groups, regions, agroecologies, and seasons.

**Fig. 1:**
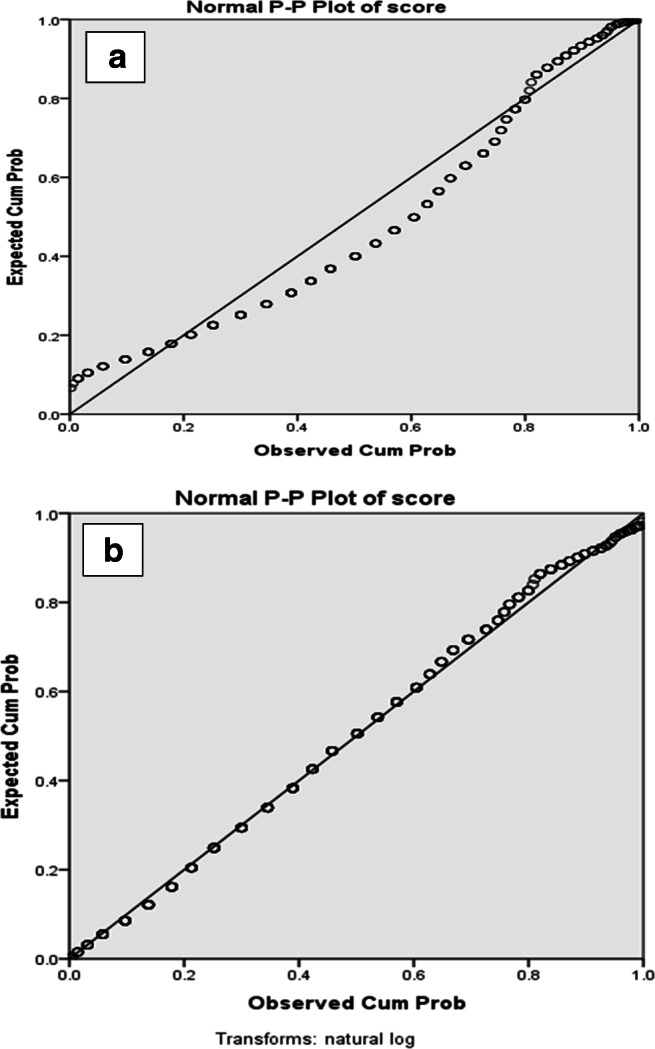
Test for normality of FGD groups’ proportional piling scores using P-P plots before (**a**) and after (**b**) natural logarithm transformation of the data

Statistical inferential analyses were employed to test the significance of farmers’ relative preferences to the different livestock species and their perceptions on the relative importance of diseases and to demonstrate the applicability of results beyond the sample respondents. Farmers’ preferences and perceptions (considered as the dependent variables) were measured as the number of beans allocated to each of the categories of the independent variables, namely the five species, diseases, four agroecologies, and seasons. The analyses were conducted using a generalized linear model procedure in SPSS version 20 (2011), fitting a natural logarithmic transformation of the data to meet the normal distribution assumption for linear model analysis. The P-P plot test showed the data distribution was normalized after transformation (Fig. 1). The odds ratio model as suggested by Abeyasekera (2001) and Agresti (1996) was fitted to compare the farmers’ preferences for the different species in reference to sheep. The interpretation of results was based on the odds of farmers preferring each species compared with sheep. The choice of sheep as a reference category was due to its high economic importance in the highlands of Ethiopia as a major source of income. Chi-squared analyses were also used for pairwise comparison of variations across regions, gender of FGD groups, agroecologies, and seasons as appropriate.

## Results and discussion

### Priority of livestock species

The majority of the focus group discussion (FGD) groups ranked cattle as their most important livestock species. The average scores allocated by men and women FGDs were 38.0 and 40.5 for cattle, 18.6 and 19.4 for sheep, 15.5 and 16.3 for poultry, 14.9 and 15.6 for goats, and 14.3 and 13.4 for equines. The scores in Amhara, Oromia, SNNPR, and Tigray regions were 41.5, 38.3, 39.3, and 37.4 for cattle, 18.5, 18.3, 19.6, and 21.4 for sheep, 12.6, 18.7, 16.6, and 16.8 for poultry, 12.9, 12.8, 16.1, and 19.4 for goat and 15.9, 15.0, 13.9, and 9.6 for equine.

The generalized linear model analysis (Table 2) indicated cattle were significantly the most preferred species with the odds of cattle scoring higher than sheep being 2.08 times (*P* = 0.000). Sheep and poultry were equally the second most important livestock, followed by goat and equine. There were no statistically significant differences between men and women FGD groups in their preference for livestock species (Table 2). There was also no significant variation in the preferences of farmers for cattle, sheep, and poultry in the four regions studied. However, goats received significantly higher scores in Tigray region compared to Oromia (odds ratio = 0.66, *P* = 0.009) and Amhara (odds ratio = 0.67, *P* = 0.005) resulting in goats being more important than poultry in Tigray. Similarly, compared with Tigray, equines were 1.65 (*P* = 0.008) and 1.55 (*P* = 0.024) times more likely to receive higher scores in Amhara and Oromia, respectively, where they are more important than goats.

**Table 2:** The odds (Exp(B)) of men and women FGD groups allocating higher scores to the different livestock species in reference to sheep in Ethiopia

	B	Std. error	Sig.	Exp(B)
Intercept	2.966	0.0655	0.000	19.414
Cattle	0.736	0.0715	0.000	2.088
Equine	− 0.370	0.1116	0.001	0.691
Goat	− 0.217	0.1068	0.042	0.805
Poultry	− 0.175	0.0974	0.072	0.839
Sheep	0^a^			1
Cattle—Men FGD	− 0.064	0.0417	0.125	0.938
Cattle—Women FGD	0^a^			1
Equine—Men FGD	0.062	0.1214	0.612	1.063
Equine—Women FGD	0^a^			1
Goat—Men FGD	− 0.048	0.1200	0.689	0.953
Goat—Women FGD	0^a^			1
Poultry—Men FGD	− 0.049	0.1037	0.636	0.952
Poultry—Women FGD	0^a^			1
Sheep—Men FGD	−0.045	0.0948	0.632	0.956
Sheep—Women FGD	0^a^			1

^a^Reference category = sheep; women FGD

The reasons provided by respondents for ranking cattle as the most important species reflected the production objectives of farmers in mixed crop-livestock systems, the most important reasons being draught power/income from fattened cattle and milk production, mentioned by 82.9% and 84.3% of the respondents, respectively. There was no difference in the reasons provided by men and women FGD groups and across the four regions, except that significantly smaller percentage of FGD groups cited milk (52.9%) and income from fattening (47.1%) in Tigray and drought power (40%) in SNNPR. Manure was also mentioned as important function of cattle by 34.3% of women and 37.1% of male FGDs, and by 43.5%, 40.0% and 41.2% of the respondents in Amhara, Oromia, and Tigray regions, respectively. Unlike in the pastoral system, socio-cultural values, and asset/saving functions of cattle were less important functions of cattle in the highland mixed crop-livestock system.

### Priority of diseases

**Fig. 2:**
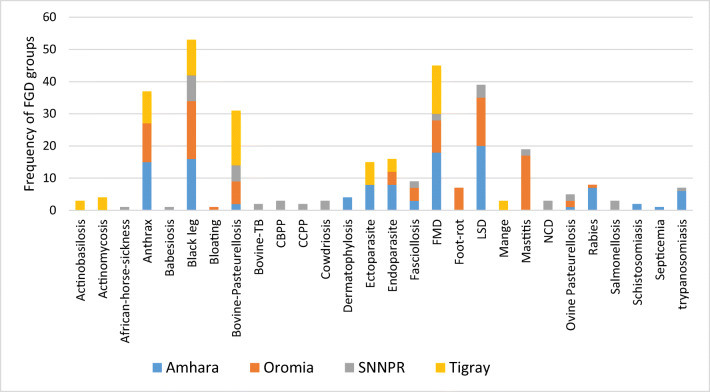
Frequency of FGD groups who mentioned 28 diseases among their top five diseases in four regions of Ethiopia. (CBPP = contagious bovine pleuropneumonia, CCPP = contagious caprine pleuropneumonia, FMD = foot and mouth disease, LSD = lumpy skin disease, NCD = New castle disease)

Twenty-eight diseases or disease syndromes were identified by the FGD groups. Reflecting the importance of cattle, most of the diseases identified were cattle diseases, though some of them also affect small ruminants. Two poultry-specific (New castle disease and salmonellosis, though salmonellosis also affects ruminants) and one equine-specific disease (African horse sickness) were also identified. However, only 12 diseases were mentioned by at least 10% of the FGD groups (Fig. 2). The 12 diseases, which were considered for further analysis, were blackleg, food and mouth disease (FMD), lumpy skin disease (LSD), anthrax, bovine pasteurellosis, mastitis, endoparasitosis, ectoparasitosis, fasciolosis, rabies, foot rot, and trypanosomiasis.

The top five diseases based on their average scores were blackleg (18.9), FMD (13.8), LSD (12.5), anthrax (10.6), and bovine pasteurellosis (8.8). Statistical analysis (Table 3) also confirmed these were the five priority diseases. Blackleg was significantly the most important disease. Farmers are more likely to allocate higher score to blackleg compared with the reference LSD (odds ratio = 1.42; *P* = 0.021). However, farmers would allocate statistically similar scores for anthrax, FMD, bovine pasteurellosis, and LSD (Table 3).

**Table 3:** The odds of farmers allocating higher scores for diseases in reference to LSD (lumpy skin disease) and men allocating higher scores in reference to women the highlands of Ethiopia

	*Β*	Std. error	Sig. (*P*)	Odds ratio
Intercept	2.593	0.1240	0.000	13.371
Anthrax	− 0.219	0.1981	0.269	0.803
Blackleg	0.351	0.1517	0.021	1.421
Bovine pasteurellosis	− 0.365	0.2175	0.094	0.694
Ectoparasite	− 1.067	0.3813	0.005	0.344
Endoparasite	− 1.186	0.4245	0.005	0.306
Fasciolosis	− 1.439	0.5375	0.007	0.237
FMD	− 0.155	0.1907	0.418	0.857
Foot-rot	− 2.105	1.0260	0.040	0.122
Mastitis	− 1.158	0.4140	0.005	0.314
Ovine pasteurellosis	− 2.511	1.5328	0.101	0.081
Rabies	− 2.071	0.9918	0.037	0.126
Trypanosomiasis	− 2.105	1.0260	0.040	0.122
LSD	0 ^a^			1
Men FGD scoring^b^				
Anthrax	0.452	0.1830	0.013	1.572
Blackleg	− 0.009	0.1240	0.942	0.991
Bovine pasteurellosis	− 0.107	0.2673	0.689	0.898
Ectoparasite	− 0.507	0.6987	0.468	0.602
Endoparasite	− 0.058	0.5914	0.922	0.944
Fasciolosis	0.018	0.7331	0.981	1.018
FMD	− 0.156	0.2227	0.483	0.855
Foot-rot	− 0.214	1.6218	0.895	0.807
Mastitis	0.047	0.5460	0.932	1.048
Ovine pasteurellosis	0.234	1.9486	0.905	1.263
Rabies	− 0.271	1.6225	0.867	0.763
Trypanosomiasis	0.728	1.1311	0.520	2.070
LSD	− 0.147	0.1898	0.439	0.863

^a^Lumpy skin disease (LSD) is the reference category. FGD focus group discussion groups

^b^The reference category: Men FGDs disease scoring were compared with women FGDs scoring for each disease

Men and women respondents had similar perceptions of diseases, their priority diseases being statistically similar except men allocated significantly higher scores to the importance of anthrax in reference to LSD (Table 3). Women and men groups’ scores were 19 and 18.3 for blackleg, 11.46 and 9.8 for FMD, 13.4 and 11.5 for LSD, 10.7 and 16.9 for anthrax, and 9.29 and 8.34 for bovine pasteurellosis. There were slight variations across the four regions in disease priorities. Among the most frequently mentioned diseases, those that were common across the regions were blackleg, FMD, LSD, anthrax, bovine pasteurellosis, and ectoparasitosis. Mastitis was mentioned in Oromia only and cowdriosis (heartwater) and Newcastle disease (NCD) in SNPPR only. In reference to the list of five top diseases across regions (Table 3), the list in Amhara and Oromia excluded bovine Pasteurellosis and included trypanosomiasis and mastitis, respectively. In SNNPR, the list included mastitis and fasciolosis and excluded FMD and anthrax. The top five in Tigray excluded LSD and ectoparasites.

The FGD groups considered a range of criteria to rank diseases (Table 4). The reasons for anthrax were the sudden death it inflicts and its potential effect on human health as it is a zoonotic disease, reasoned by 81.2% and 50.0% of the FGDs. Although not supported by the existing modern veterinary knowledge, 7.1% of the respondents considered LSD as a zoonotic disease. Blackleg was perceived to infect animals with good body condition such as well fattened animals. LSD was associated with high morbidity rate resulting in reduced milk production and work performance of oxen by almost all respondents and high transmission rate by about a third of the respondents who ranked the disease.

**Table 4:** Percentage of farmers providing reasons for top ranking diseases in the highlands of Ethiopia

Reasons for ranking	Anthrax	Blackleg	Bovine pasteurellosis	FMD	LSD
Zoonotic disease	50.0	6.2	0.0	0.0	7.1
Affects all age groups/species	43.8	6.2	0.0	75.0	14.3
Affects animals in good condition	6.2	56.2	100.0	75.0	0.0
Sudden death	81.2	37.5	66.7	0.0	7.1
High morbidity/effect on productivity	0.0	37.5	0.0	25.0	92.9
High mortality	43.8	18.8	0.0	25.0	7.1
High transmission rate	0.0	12.5	0.0	25.0	35.7
Affects skin quality	0.0	0.0	0.0	0.0	21.4
Not common in locality	0.0	6.2	0.0	0.0	0.0

### Agroecological and seasonal distribution of diseases

Agroecology is the major determinant of disease epidemiology. Based on a combined criterion of the proportion of respondents ranking the diseases 1st to 3rd and those not including the disease in their top five list, Anthrax, FMD, and bovine pasteurellosis were the most important diseases in the dry highlands (Table 5). LSD and blackleg in the moist lowlands and blackleg and LSD in the wet highlands were the most important diseases.

**Table 5:** Per cent of respondents ranking the top five diseases in different agro-ecological zones of Ethiopia

Agroecology ^1^	Rank ^2^	Anthrax	Blackleg	Bovine pasteurellosis	FMD	LSD
Dry highland	0 ^3^	38.1^a 4^	38.1^a^	28.6^a^	19.0^a^	90.5^b^
	1	38.1^a^	14.3^a, b^	14.3^a, b^	19.0^a, b^	–
	2	19.0^a, b^	9.5^a, b^	42.9^b^	9.5^a, b^	–
	3	4.8^a^	23.8^a^	4.8^a^	23.8^a^	–
Moist highland	0	46.9^a, b^	12.5^c^	78.1^b^	28.1^a, c^	25.0^a, c^
	1	21.9^a, b^	18.8^a, b^	–	–	34.4^a^
	2	28.1^a^	31.2^a^	12.5^a, b^	–	9.4^a, b^
	3	–	15.6^a, b^	9.4^a, b^	28.1^b^	9.4^a, b^
Wet highland	0	56.2^a, b^	31.2^a, b^	62.5^a, b^	75.0^b^	18.8^a^
	1	6.2^a, b^	43.8^b^	–	–	18.8^a, b^
	2	–	25.0^a^	–	–	18.8^a^
	3	12.5^a^	–	–	6.2^a^	–

^1^Result for moist lowland zone is not presented. None of the respondents included anthrax and LSD in the top five diseases list, whereas all respondents in this zone ranked Blackleg, pasteurellosis, and FMD as the 2nd, 4th, and 5th most important diseases, respectively

^2^Ranks derived from the number of beans (scores) allocated to the diseases

^3^The disease was not included in farmers’ lists of top five diseases

^4^Different superscript letters within row denotes percent of respondents ranking the diseases differ significantly from each other at the 0.05 level

**Fig. 3:**
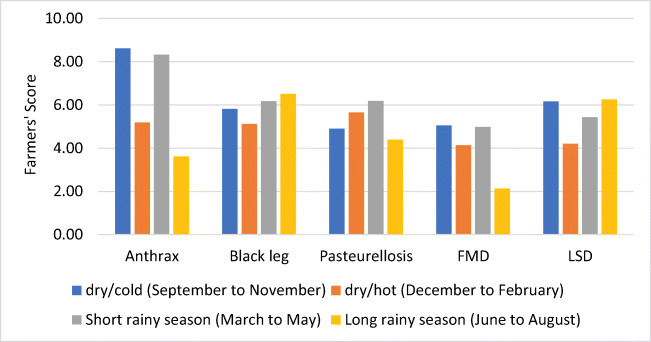
Average scorings of farmers for the prevalence level of five top diseases in four seasons of the year in the highlands of Ethiopia

Seasons of the year in the highlands of Ethiopia are generally divided into four, although there are some variations across agroecological zones and geographic locations (regions). Generally, the four seasons are the dry/cold (September to November, dry/cold), the dry/hot (December to February, dry/hot), the short rainy (March to May, short rainy season), and the long rainy (June to August, long rainy season) seasons. Interviewed farmers observed a higher prevalence of anthrax in dry/cold and short rainy seasons, blackleg in season dry/cold, short rainy seasons, and long rainy seasons, pasteurellosis in season dry/hot and short rainy seasons, FMD in dry/hot and short rainy seasons, and LSD in dry/cold season and long rainy seasons (Fig. 3).

### Transmission and impacts of diseases

Women and men respondents’ understanding of disease transmission pathways was similar. Adults were more responsible than the rest of the family members for the transmission of diseases. Adult men played more role in the transmission of non-zoonotic diseases, but both men and women were equally responsible for zoonotic diseases (Table 6). Adult men scored more for transmission activities involving animal movement, disposal of dead animals, and handling of contaminated tools, whereas women scored more in activities related to managing sick animals. However, both men and women are equally responsible for the transmission of zoonotic diseases.

**Table 6:** Respondents’ scorings (out of 20) of the roles of family members in the transmission of diseases in the highlands of Ethiopia

Transmission activity	Adult men	Adult women	Young males	Young females	Children
*Non-zoonotic diseases*					
Moving animals to distant places	11.0	0.0	9.0	0.0	0.0
Burying dead animals	10.0	4.0	4.7	1.3	0.0
Handling contaminated tool	14.0	6.0	0.0	0.0	4.0
Eating/drinking raw meat/milk	9.0	2.3	2.3	2.3	4.0
Eating without washing hands	4.0	6.7	2.7	5.0	1.7
Feeding	12.0	6.7	0.3	1.0	0.0
Watering	6.0	4.0	4.5	2.2	3.2
Herding	7.3	3.4	6.0	2.3	0.8
Housing animals with humans	4.0	4.0	4.0	4.0	4.0
Exposing to insect bite during herding	10.3	3.8	3.5	1.8	1.8
Managing sick animals	6.5	7.8	2.3	3.2	0.2
Marketing	9.2	4.7	5.8	0.6	0.0
Assisting mating	7.0	12.0	0.0	1.0	0.0
Agricultural activities	15.7	0.0	4.3	0.0	0.0
General farm work (livestock + agriculture)	8.7	5.8	2.5	1.7	1.3
Overall	9.0	4.7	3.5	1.8	1.4
*Zoonotic* diseases					
Cleaning Barns	0.0	6.0	6.0	8.0	0.0
Eating/drinking raw meat/milk	6.8	4.8	3.0	2.3	2.0
Feeding	5.0	10.0	1.0	4.0	0.0
Handling contaminated feed	3.0	13.0	2.0	2.0	0.0
Herding	5.6	4.5	4.1	2.3	3.4
Watering	4.1	5.1	3.6	2.8	4.4
Housing animals with humans	7.0	4.0	6.0	3.0	0.0
Managing sick animals	10.8	6.0	0.8	2.0	0.0
Marketing	9.8	4.6	5.0	0.6	0.0
Assisting mating	10.0		10.0		
Slaughtering	6.0	4.2	1.0	0.6	8.2
Slaughtering or cooking	9.2	4.2	4.2	3.0	0.0
Renting animals form neighbors	9.0	6.5	4.5	0.0	0.0
Treating sick animals	13.4	4.5	4.4	0.8	1.0
General farm work (livestock + agriculture)	6.5	9.0	2.5	2.0	0.0
Overall	7.1	6.2	3.9	2.4	1.4

The major effects of diseases were loss of household income, resulting from lower animal productivity and higher mortality, and zoonotic risks (Table 7). Anthrax was recognized by 77.5 of the respondents as the most important zoonotic disease. Loss of oxen and other valuable animals could also impact agricultural activities and saving/insurance since livestock serve as capital store in rural Ethiopia. In extreme cases, farmers could be displaced from their ancestral land and migrate to towns or become dependent on government handouts and children would be out of school.

**Table 7:** Percentage of FGD groups reporting the different impacts of diseases on households in Ethiopia

Impacts	Anthrax	Blackleg	Bovine pasteurellosis	FMD	LSD	Overall
Reduced income	77.5	69.8	83.3	81.7	73.2	76.7
Mortality	45.0	55.6	50.0	51.7	39.0	49.2
Reduced productivity	42.5	61.9	72.2	65.0	73.2	62.9
Time for caring sick animals	12.5	12.7	13.9	8.3	9.8	11.2
Cost of treatment	22.5	15.9	8.3	11.7	19.5	15.4
Reduced market value	10.0,	7.9	–	3.3	34.1	10.4
Malnutrition	10.0	14.3	2.8	20.0	9.8	0.12
Impact on human health	77.5	14.3	19.4	–	14.6	22.1
School drop outs	25.0	7.9	–	18.3	9.8,	12.5
Psychological/social impacts	–	6.4	5.6	1.7	2.4	0.84
Renting-out land/renting-in oxen due to loss of oxen	20.0	7.9	2.8	18.3	12.2	12.5
Migration	15.0	9.5	–	18.3	9.8	11.2

**Fig. 4:**
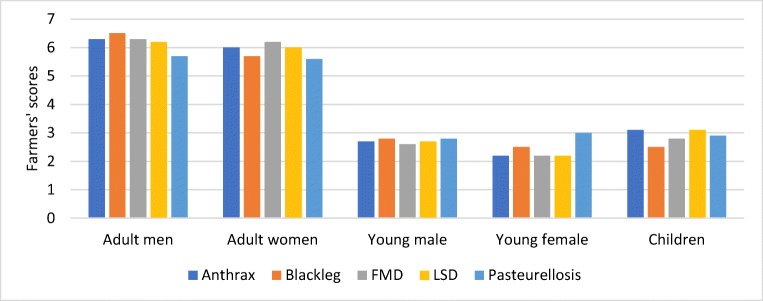
FGD groups’ scoring (out of a total score of 20) of the impacts of diseases on family members in the highlands of Ethiopia

Women and men adult members were equally more affected by diseases than the other members of the family (Fig. 4), the reason provided by the respondents being that adults had to work harder to supplement their reduced income and animal source foods due to death of oxen and milking cows. Adult members were also more at risk of zoonotic diseases, as they were more responsible for caring, slaughtering, and cooking meat from animals unsuspected of being infected with zoonotic diseases. However, the perception of the FGD groups varied. Twenty-eight percent of FGD groups believed lower livestock productivity and higher mortality would affect women more since they are directly responsible for providing food for the family including the husband and care for young children who depend on cow milk. Women are also more affected by zoonotic diseases as they are responsible for the management of sick animals. The rest provided either equal (24.9% of FGDs) or more scores to male adults (46.9% of FGDs).

Male and women farmers had similar understanding of the effects of diseases on the different classes of animals (Table 8). Farmers understood that anthrax affect all age groups of livestock but inflicts high morbidity in adults. Incidence of anthrax was low in young animals up to the age of 3 months as they were kept indoor and less likely to be exposed to the pathogen. However, young grazing animals aged 3–12 months and animals in good body condition/with heavy muscle were severely affected. LSD and blackleg were also understood by farmers to affect mostly adult animals, as they are exposed to the external environment, and animals in good body condition in the case of blackleg. On the contrary, the farmers observed that FMD occurs in all age groups, but it is very severe among younger age groups (calves) due to their low ability to cope with the disease.

**Table 8:** Animal age groups most affected by five most important diseases as perceived by women and men farmers in Ethiopia

		Scorings of women and male FGD groups			
Disease	Gender of FGD members	Newborn	Young	Adult female	Adult male
Anthrax	Men	2.3	8.2	10.5	14.1
	Women	4.7	7.5	11.3	14.5
Blackleg	Men	2.9	6.3	11.0	14.6
	Women	1.3	5.9	12.1	19.3
Bovine Pasteurellosis	Men	0.0	12.0	12.0	16.0
	Women	0.0	8.0	12.0	18.0
FMD	Men	14.7	4.5	9.0	8.5
	Women	14.7	4.5	9.0	8.5
Lumpy skin disease	Men	1.6	4.6	12.1	13.3
	Women	3.7	6.0	12.1	13.9

*FGD* Focus group discussion, *FMD* foot and mouth disease

### Disease coping mechanisms

Farmers coping mechanisms against diseases include both traditional and modern practices. The traditional practices include treating sick animals with herbs such as *Endod/Phytolacca dodecandra* against anthrax, incision on the brisket and mandibular vein of animals affected with anthrax, incision on the hind quarter, and burning the affected part with hot metal/branding in the case of blackleg, use of ‘holy water’, applying crushed millipedes mixed with honey on the tongue of animals affected with FMD. There are also traditional superstitious practices. The modern practices mentioned were treatment of sick animals in veterinary clinics, vaccination, and health management practices including isolation of sick animals and improved feeding and housing management for sick animals. Most of the farmers used modern treatment and a combination of modern and traditional treatment (Table 9). Improved health management was the least practiced intervention.

**Table 9:** Percentage of respondents using different coping mechanisms against five top livestock diseases in Ethiopia

Coping mechanisms	Anthrax	Blackleg	Bovine pasteurellosis	FMD	LSD	Overall
A *	23.1^a, b^	27.6^a, b^	52.8^b, c^	16.9^a^	65.9^c^	34.80
B	7.7^a^	–	2.8^a^	8.5^a^	9.8^a^	5.60
A + B	7.7^a^	6.9^a^	2.8^a^	8.5^a^	9.8^a^	7.30
C	–	3.4^a, b^	5.6^a, b^	18.6^b^	2.4^a, b^	6.90
A + C	51.3^a, b^	50.0^b^	19.4^c^	23.7^a, c^	9.8^c^	31.80
A + C + B	2.6^a^	12.1^a^	13.9^a^	5.1^a^	2.4^a^	7.30
C + B	7.7^a, b, c^	–	2.8^a, b, c^	18.6^b^	–	6.40

*^a^Modern veterinary services, including vaccination and treatment in veterinary clinics

^b^Health management, including isolation of sick animals, feeding, and housing of sick animals,

^c^Traditional healing, incisions, branding, use of herbs, and some superstitious ones

## Discussion

Livestock play multiple roles in the livelihoods of smallholder farmers in the highlands of Ethiopia. Thus, most farmers keep all types of livestock species since each species serves a different function and such relative importance of livestock species determines farmers’ relative preferences for the different species. The preference for cattle over other species in the current study is to be expected. In the highland mixed crop-livestock system, cattle play a unique role besides providing animals and animal products for home consumption and sale, namely providing oxen power. This was also confirmed by 82.9% respondents in the current study who mentioned drought power as their reason for prioritizing cattle over other species. Sickness or death of oxen could result in extra costs for renting oxen, or worse, having to renting out crop land and could lead to absolute poverty. Land holding and ownership of oxen are among the significant determinants to avoid rural poverty in Ethiopia (Bogale et al. 2005). Accordingly, most of the diseases identified by the FGD groups in the current study were cattle diseases including two diseases among the top five diseases affecting cattle only, emphasizing the role of healthy cattle.

The livestock health situation in Ethiopia is comparable or worse than in other African countries. Across Africa, disease outbreaks have been increasing. For instance, FMD (and also pasteurellosis) outbreaks have been increasing from 378 outbreaks from 26 countries in 2009, to 454 outbreaks from 24 countries in 2010, and to 902 outbreaks from 28 countries in 2011. A similar trend was also reported for pasteurellosis. In 2011, Ethiopia reported the highest number of FMD fatalities (721 = 0.0009% of the ruminant population), the highest number of pasteurellosis outbreaks (570 = 0.00071%), and the highest number of deaths from pasteurellosis (1633 = 0.002%) among the AU-IBAR countries (http://www.au-ibar.org/foot-and-mouth-disease-in-ruminants).

The priority diseases identified by the FGDs in the current study correspond to previous findings in the same regions. Dereje and Shibiru (2016) reported that out of 1632 examined disease outbreaks from 2008 to 2013 in 17 districts in East Wollega zone of Oromia region in Ethiopia (one of the current study sites), 21.8%, 21.6%, 15.6%, 9.3%, 7.8%, and 7.2% were found to be positive for bovine pasteurellosis, blackleg, LSD, FMD, CBPP, and anthrax. Similar disease priorities have been reported from SNNPR region (blackleg, mastitis, lumpy skin disease, and tick infestation; Albe et al. 2016) and Tigray region (blackleg, anthrax, LSD, mastitis, and FMD in order of importance; Tekle 2007). Pastoralists and farmers traditionally describe livestock diseases by their local names, and some of the diseases are locally known by the same name in more than one region, though the communities in the different regions speak different languages. For instance, blackleg (*Gangraena emphysematosa* caused by *Clostridium chauvoei*) is known as *Aba Gorba* both in Oromia and SNNPR, and bovine pasteurellosis as *Gororsa* in Amhara, Oromia and SNNPR regions. Local veterinarians are well acquainted with the local disease nomenclatures which help to translate farmers perceptions into modern veterinary nomenclatures.

According to Dereje and Shibiru (2016), lack of attention for prophylactic vaccination, misuse of veterinary drugs, lack of proper management, and poor outbreak reporting system could have contributed to the high prevalence of some of the diseases (bovine pasteurellosis, blackleg, and LSD). Similarly, a review of animal health service in the four highland regions of Ethiopia (similar to the location of the current study), Hooper (2016) has shown that most of the animal health services are provided by the government, while the private sector involvement is insignificant mainly serving as drug shops. The network of government animal health service is well structured with rural animal health posts serving a group of two to five *kebeles* and district level clinics coordinating the rural health posts and diagnostics and surveillance laboratories at region level. However, while the district clinics are relatively well staffed and equipped, the rural health posts are often ill equipped and staffed, and the situation is challenging for the rural staff to meet their responsibilities. Further, the regional laboratories are not functioning at their full capacity or not meeting their goals.

The relative importance of diseases would vary depending on the criteria used for ranking or when ranking is done from different perspectives. For instance, the livestock sector analysis for the livestock master plan of Ethiopia (Shapiro et al. 2017) ranked livestock diseases based on three criteria, namely the impact on households and livelihood framework, impact on markets and value chains, and impact on intensification pathways in the production systems. The scores provided using the three criteria were again weighted, respectively, according to the share of the households in the affected production systems, total value added generated from the sub-chain affected, and the animal population in production systems affected. Among the top 10 diseases ranked based on their incidence, the top three livestock diseases according to the three criteria were, respectively, FMD, CBPP, and tuberculosis; FMD, LSD, and brucellosis; and brucellosis, FMD, and tuberculosis. Ranking of diseases may also vary depending on the category of respondents. These priority diseases included the priority diseases of the farmers in the current study but included other diseases. Priority of diseases could also differ depending on the respondents. Hassen et al. (2014) interviewing smallholders and professionals in North Gondar, Amhara region, found LSD as the most important disease for smallholder farmers, being mentioned by 46.3% of the respondents, while anthrax and gastro-intestinal tract parasites were more frequently mentioned by professionals in the public and private services, and FMD was frequently mentioned by both professionals and farmers.

Livestock disease priorities may vary depending on various determinants. In the current study, variations were found in diseases women and men farmers prioritize, and variation in the relative importance of diseases across agro-ecological zones and seasons. Livestock development projects commonly target the male household heads for identifying constraints and designing development strategies. The approach followed in the current study, namely separate FGDs with both women and men groups in the same *kebele*, enabled to assess the perceptions and priorities of both women and men farmers, providing a more in-depth view of disease importance. The results showed that women may have similar perceptions, understanding, and priorities as men, though there are some differences between the genders including slight variation in their perceptions regarding the relative importance of anthrax. We found that agroecology and seasons determine the epidemiology of diseases. The high importance of LSD in the wet highlands in the current study is supported by the characteristics of this disease to be closely associated with prolonged and heavy rains which favor an increase in the population of the vector (Regassa 2003). Similarly, LSD was reported in Uganda by 67.5% of respondents in semi-humid tropics (above 1000 mm) and 86.1% in warm humid tropics (1000–1500 mm) but only by 20% in warm semi-arid climate receiving 500–1000 mm annual rainfall (Ocaido et al. 2009).

Farmers associate disease incidence with seasons. The high incidence of anthrax during the drier seasons compared with the long rainy season was, according to respondents’ reasoning, due to the high temperature and feed shortage which forced the animals to graze close to the ground and contract the disease agents. Farmers associated blackleg, although it occurs throughout the year, with wet seasons (long and short rainy seasons) where feed is more available and body condition of the animals are improved and are more vulnerable to the disease. The respondent farmers seem to be highly knowledgeable about blackleg epidemiology which corresponds to the epidemiology of clostridial diseases (PRIMEFACT 2007) in that it generally affects the best conditioned animals, with most losses occurring during wet seasons when clostridial spores are washed out and where there is an abundance of feed. Farmers associate LSD with the wet season as a result of increased stagnant water bodies and contact between different village herds in communal grazing lands. FMD is believed to occur when animals are in free range during the months of October to January. Bovine pasteurellosis is mostly associated with seasons of hot weather (February to May) and workload such as plowing.

The use of participatory epidemiology tool for disease surveillance, survey, prioritization, and control has been increasing, especially since 2012 in Africa and Asia, according to a comprehensive inventory of participatory epidemiology activities (Alberto  et al. 2017). Unlike the criteria used to rank diseases in conventional studies which rely mainly on incidence or prevalence of diseases (Hooper 2016; Shapiro et al. 2017), farmers consider a range of criteria to rank diseases. For instance, in the current study, the main underlying reasons mentioned by the respondents for allocating the highest scores the suddenness of death, zoonosis, and the types of animals the disease affects. For instance, blackleg was ranked high since it infects animals with good body condition such as well fattened animals which could fetch tens of thousands of Birr (the local currency) and thus cause great economic loss for households affected by the disease. Farmers also consider the diverse function of cattle in ranking diseases such as effect of diseases on work performance of oxen, as was the case in the current study in ranking LSD, which may not be considered in conventional studies. Proportional piling rather than direct ranking was used in the current study. Proportional piling is a suitable approach for working with illiterate farmers as it is a visual assessment tool. Its advantage over ranking is that the results are numerically meaningful providing a distance measure between preferences for different items. The limitation of the approach could be forced scoring when small number of seeds/pebbles/beans are used. However, this shortcoming can be overcome by using a larger number (100) of seeds/pebbles/beans since this increase respondents’ flexibility in expressing their strength of preference for one item over another (Abeyasekera 2001).

A striking similarity is observed between the ranking of diseases by farmers in the current study and by clinicians and heads of district offices of livestock agencies in a previous study in the same four regions (Hooper 2016) where all the five top diseases from the current study were indicated as the most common diseases except trypanosomiasis which was mentioned by more professionals (33) than FMD (27). This confirms that farmers are knowledgeable about diseases. A similar argument has been made that local farmers are knowledgeable about common disease that affect their poultry, have good diagnostic abilities, and can recognize clinical signs, and recommendations have been made that there should be improved veterinary outreach to share livestock health information through active community participation, and participatory epidemiology should be incorporated into state and national disease surveillance systems (Jibril et al. 2015). Indigenous disease treatment knowledge and practices identified through participatory epidemiology need to be investigated and utilized as alternative remedies. For instance, application of hot water or hot iron on the affected portion of animals infected with blackleg in the current study is shared by farmers in other parts of Africa (Mahachi 2016). Nevertheless, the current study also indicated gaps in farmers’ knowledge of the epidemiology of diseases. For instance, some FGD groups considered FMD and LSD as zoonotic diseases and superstitious practices were reported as remedies for diseases.

The high incidence of diseases reported by the FGDs in this study could be attributed to the veterinary service situation in the country. According to the 2013 animal health strategy and vision for Ethiopia (MoA and ILRI 2013), growth of private animal health service delivery is constrained by absence of an enabling policy environment, a system of sanitary mandates does not exist, only 45% of the country is served by the animal health delivery systems. Although the National Veterinary Institute is producing a wide range of vaccines, some essential vaccines are not produced or are not produced in sufficient quantity and quality to achieve sufficient vaccination cover to interrupt disease transmission, and disease surveillance and reporting is poor and irregular, with only about 30–35% of the districts submitting disease outbreak reports (MoA and ILRI 2013).

## Conclusions

Cattle are the most important livestock for smallholder farmers in the highlands of Ethiopia. The top five diseases afflicting cattle in the highlands of Ethiopia are blackleg, anthrax, LSD, FMD, and bovine pasteurellosis. However, the relative importance of diseases varies across agro-ecological zones and seasons. This study confirmed that men and women farmers have similar and equal understanding of the epidemiology of diseases and are equally affected by diseases. It is therefore commendable to include both male and female household members in designing health interventions. Farmers use both modern and alternative traditional remedies to prevent diseases and treat sick animals. However, vaccination and health management are rarely used. The PE research approach used in this study proved to be reliable approach to get insights into farmers’ knowledge of disease epidemiology, which corresponded to a large extent to results from conventional studies. Based on the findings of this study, the intervention strategies listed in the 2013 animal health strategy (MoA and ILRI 2013) seem pertinent and need to be implemented to overcome the challenges of diseases in the country.
