# Correction to: Importance of livestock diseases identified using participatory epidemiology in the highlands of Ethiopia

**DOI:** 10.1007/s11250-021-02593-7

**Published:** 2021-02-17

**Authors:** Solomon Gizaw, Hiwot Desta, Biruk Alemu, Azage Tegegne, Barbara Wieland

**Affiliations:** International Livestock Research Institute, PO Box 5689, Addis Ababa, Ethiopia

**Correction to: Tropical Animal Health and Production (2020) 52:1745–1757.**

10.1007/s11250-019-02187-4

The article “Importance of livestock diseases identified using participatory epidemiology in the highlands of Ethiopia”, written by Solomon Gizaw, Hiwot Desta, Biruk Alemu, Azage Tegegne, and Barbara Wieland, was originally published Online First without Open Access. After publication in volume 52, issue 4, page 1745–1757 the author decided to opt for Open Choice and to make the article an Open Access publication. Therefore, the copyright of the article has been changed to © The Author(s) 2020 and the article is forthwith distributed under the terms of the Creative Commons Attribution 4.0 International License, which permits use, sharing, adaptation, distribution and reproduction in any medium or format, as long as you give appropriate credit to the original author(s) and the source, provide a link to the Creative Commons license, and indicate if changes were made. The images or other third party material in this article are included in the article’s Creative Commons license, unless indicated otherwise in a credit line to the material. If material is not included in the article’s Creative Commons license and your intended use is not permitted by statutory regulation or exceeds the permitted use, you will need to obtain permission directly from the copyright holder. To view a copy of this license, visit http://creativecommons.org/licenses/by/4.0/.

The original article has been corrected.

